# Transversely isotropic elastic-plastic properties in thermal arc sprayed Al–Zn coating: a microporomechanics approach

**DOI:** 10.1038/s41598-020-67694-z

**Published:** 2020-07-07

**Authors:** Wai Yeong Huen, Hyuk Lee, Vanissorn Vimonsatit, Priyan Mendis, Han-Seung Lee

**Affiliations:** 1 Civil and Mechanical Engineering, Curtin University, Bentley, WA 6102 Australia; 2 Faculty of Science and Engineering, Macquarie University, Sydney, NSW 2109 Australia; 3 Infrastructure Engineering, University of Melbourne, Parkville, VIC 3010 Australia; 4 Architectural Engineering, Hanyang University, Ansan, Korea

**Keywords:** Civil engineering, Characterization and analytical techniques

## Abstract

The transversely isotropic behaviour of thermal sprayed aluminium and zinc coating has been investigated based on a combination of nanoindentation experimental data and microporomechanics theory. A recently developed strength homogenisation approach comprises of the solid and porous medium is adopted to investigate the morphology properties of thermal sprayed aluminum and zinc coating. The finding of this paper demonstrates that the individual aluminum and zinc phases in the coating have a characteristic packing density close to the theoretical highest spherical packing ratio for face-centred cubic and hexagonal close packed. Also, the plasticity properties of solid particles in both aluminum and zinc are found to have a significant transversely isotropic condition, while the elasticity properties are close to isotropic. These findings led to the conclusion that the anisotropic condition of the coating is dominantly affected by the plasticity properties, in terms of cohesion and friction coefficient.

## Introduction

The thermal sprayed coating is often referred to a group of coating consists of either metallic or non-metallic coating that are designed to provide corrosion and wearing protection, and in some cases protection against high temperature exposure to the underlying substrate^1,2^. Investigating the coating mechanical properties is paramount to improve its performance and reliability in engineering application. In recent years, nanoindentation has been used extensively to investigate the mechanical properties of the thermal sprayed coating materials due to its ability to measure material responses at the microscale^3–6^. When coupled with statistical indentation method, nanoindentation is capable of providing insight into the mechanical behaviour of heterogeneous material such as thermal sprayed composite coating that has complex microstructures and multi-phase components interactions^4,7,8^. In particular, nanoindentation has been used effectively to investigate anisotropic properties in the coating as a result of the deposition nature of the interlamellar microstructures that give rise to the directionally dependent mechanical behaviour^9,10^. Recent literature found that the nanostructured pores of high-performance thermal sprayed coating are related to the mechanical behaviour in crack propagation resistance and wearing capacity^11–13^. However, research showing the relationship between the nanostructured porosity and the microscale mechanical properties such as elastic modulus and hardness remains scarce. It should be noted that the nanostructured pores^11,12^ are the pores between the grain’s boundary (area equal or less than 300 nm). In this paper, the pores at the boundary of the grains will be derived analytically at the microscale level and therefore they are referred to as microporosity from here on.

The primary aim of the present work is to investigate and quantify the microporosity in thermal sprayed aluminum–zinc (Al–Zn) coating using a combination of microporomechanics theory and nanoindentation measured data. In this approach, the thermal sprayed coating is represented by a heterogeneous material model consists of self-consistent and perfectly disordered solid granular particles^14–16^. As a result, the microporomechanics theory can be used to predict the elasticity and plasticity parameters of the granular particles in the material model, which are presented as the outcome in this paper. The focus of this paper is to present the downscaling methodology that enables the correlation of the nanoindentation data carried out at microscale^17^ to derive the transversely isotropic mechanical properties of the nanoscale solid granular particle. The purpose of doing so is to enable future work to be carried out on multiscale homogenisation modelling^18,19^, where the solid particle’s mechanical parameters are the input values. However, the homogenisation process to provide the correlation between the microscale to the macroscale is not part of the scope in this paper.

This paper is presented in the following sequence. The first part of this paper describes the coating sample preparation and nanoindentation experiment setup, followed by the explanation of how the nanoindentation data is acquired by using a combination of dimensional analysis and artificial neural network. Subsequently, the simplified material model is presented that forms the foundation of the microporomechanics theory. This is followed by the presentation of micromechanics theory and the downscaling algorithm used to determine the mechanical parameters of the solid particle in the material model. Finally, the microporosity correlation with nanoindentation elastic modulus and hardness is tabled, and the outcome of the downscaling algorithm is discussed.

## Experiment

**Figure 1:**
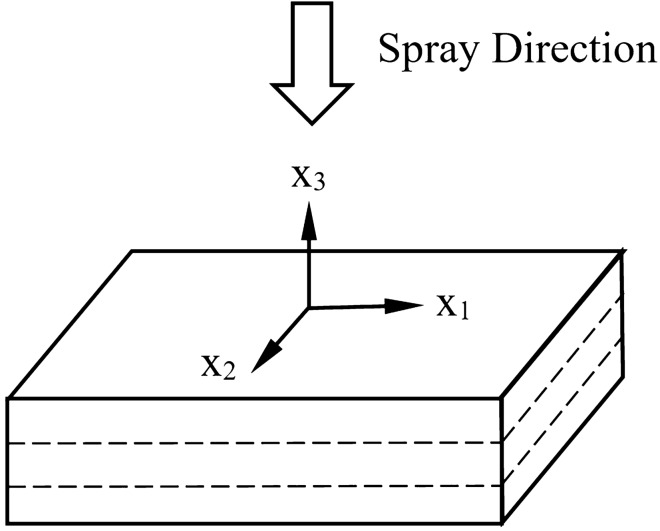
Transversely isotropic model with a transversely isotropic plane ($x_1$–$x_2$) and an axis of symmetry ($x_3$).

Nanoindentation is carried out on thermal arc sprayed aluminum-zinc coating that has been applied on mild steel substrate using commercially pure aluminum and zinc (99.95 wt.%) wires as feedstock. The applied coating has a thickness at approximately 100 microns and cast with resin on the coating surface to fill up any voids. The sample is then polished following standard ASTM E3-11 with subsequent additional polishing to reduce the surface roughness further. Nanoindentation is carried out using the standard XP CSM method with a Berkovich tip. A total of approximately 500 nanoindentation locations has been carried out. This work follows a similar approach from previous work^20^ to model the coating as a transversely isotropic model with elasticity and plasticity properties as shown in Fig. 1.

## Data acquisition

**Figure 2:**
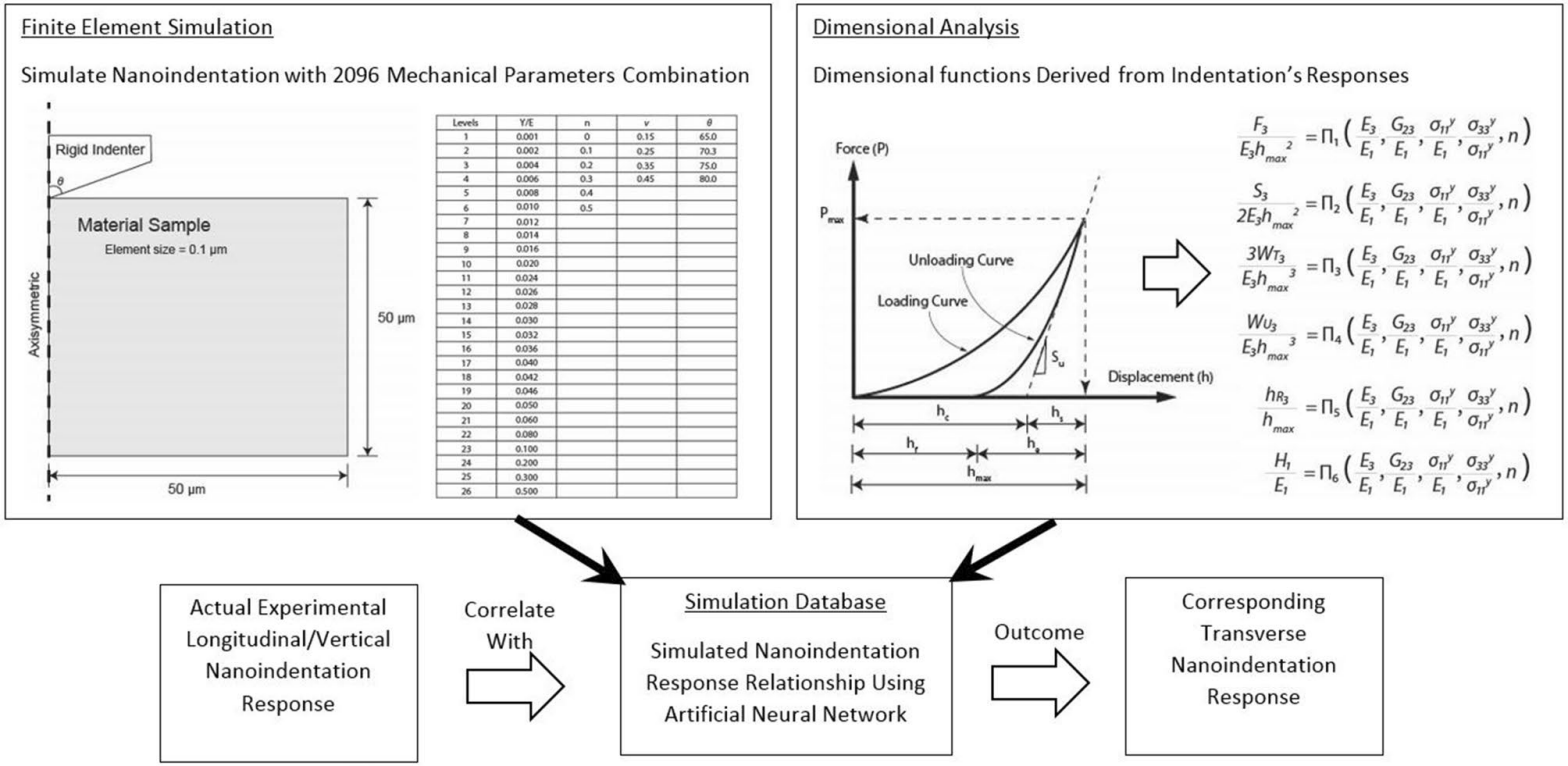
Flow chart showing derivation of transverse properties using a combination of finite element simulation, dimensional analysis and artificial neural network.

Existing researches^5,21^ showed that indentation measurement has implicit response parameters derived from the load and unload curve from nanoindentation which can be inter-related using the dimensional function approach based on the work by Cheng and Cheng^22^. Conventionally, the indentation modulus (*M*) and hardness (*H*) results are calculated from this force and displacement response using the popular Oliver–Pharr method ^23,24^. However, it has been shown that the Oliver-Pharr method can only provide estimation based on the elastic and isotropic condition^25^. To address these gaps, this work extends the methodology demonstrated in previous works ^5,21^ by using a combination of dimensional analysis, finite element simulation and artificial neural network. The simulated indentation data in both directions are firstly organised into respective dimensional functions, and their relationship are later compiled using artificial neural network (ANN) with commercial computation analysis software^26^. The measured experimental nanoindentation data is then correlated with the machine learning database using a nonlinear solver to obtain the corresponding transverse direction elastic and plastic mechanical properties. The outcome of this approach enables the entire transversely isotropic model mechanical properties to be described with six parameters, i.e. longitudinal elastic modulus ($E_3$), transverse elastic modulus ($E_1$), longitudinal yield strength (${\sigma _{33}}^y$), transverse yield strength (${\sigma _{11}}^y = {\sigma _{22}}^y$), shear modulus ($G_{23}$) and work hardening coefficient (*n*). Based on the relationship determined using the artificial neural network, the transverse properties can be subsequently determined using the actual experimental longitudinal nanoindentation result. This process is summarised in a flow chart presented in Fig. 2.

## Microstructure model

Thermal arc sprayed coating is formed by staking and solidification of molten metal particles on the substrate, propelled by spray torch as a result of melting metal electrodes by an arc using a high current power source^27^. The quality of the coating is dependent on how the propelled solid metal particles (after cooling down) to form a splat formation that bond with each other and on to the substrate^28^. The deposited solid particles’ microstructure and their mechanical properties are expected to differ from the virgin bulk solid-state particles in wire form due to plastic deformation, exposure to high heat-intensive and kinetic pressure during impact^29,30^. As a result, the microporosity within the grain structure would be different when compared to the state in wire form. In this work, a homogenised porous medium consists of both the grain (solid particle) and the microporosity in between each solid particle. The packing density parameter represents the volume ratio of the solid over the microporosity. Identification of microporosity effect on the mechanical properties measured from nanoindentation in this homogenised medium arrangement has been extensively studied in other heterogeneous materials such as concrete^31^ and shale^32^, which is adopted to describe the thermal sprayed composite coating mechanical properties in this work.

**Figure 3:**
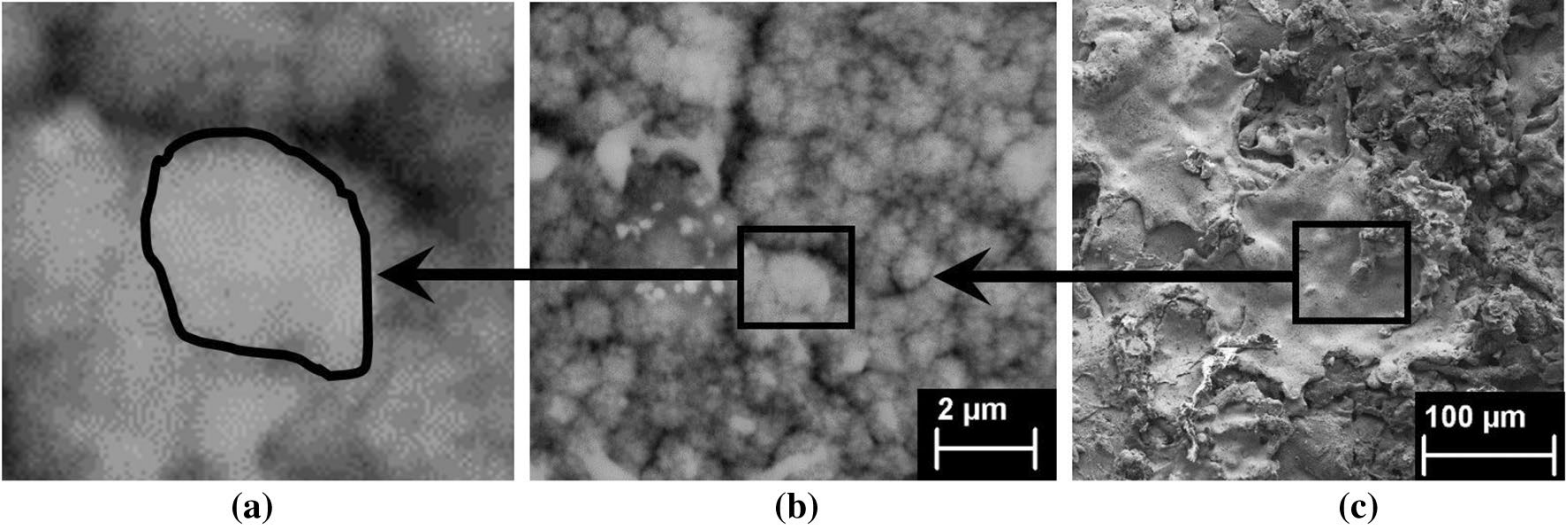
Figure 1 SEM images from the thermal arc sprayed composite (aluminum and zinc) coating. (**a**) Level 0 Nanoscale solid particle (**b**) Level I Microscale solid-porous structures with microporosity between adjacent solid particles (**c**) Level II Macroscale microstructure showing the molten splat with cracks and pores.

The thermal sprayed composite coating is shown here in three distinct characteristic length scales referred to as Level 0, Level I and Level II (see Fig. 3). Level 0 refers to the elementary solid particle that takes the form of a single grain with a mean size of 0.25–$0.5\,\upmu \hbox {m}$^1^. Level I refers to the composite microscale solid-porosity structure where pores can be observed around the grain boundary of solid particles as a result of temperature changes and kinematic impact^28^. At this scale level, the morphology structure is represented by individual splat deposition that generally ranges between 10 and $50\, \upmu \hbox {m}$^1^. The pores between the solid particles at this scale level are the microporosity measured by the packing density ($\eta$). The next level up is the macroscale level (Level II) where the coating microstructure is characterised by a combination of interlamellar splat deposition with cracks network and defects^28,33,34,34^.

**Figure 4:**
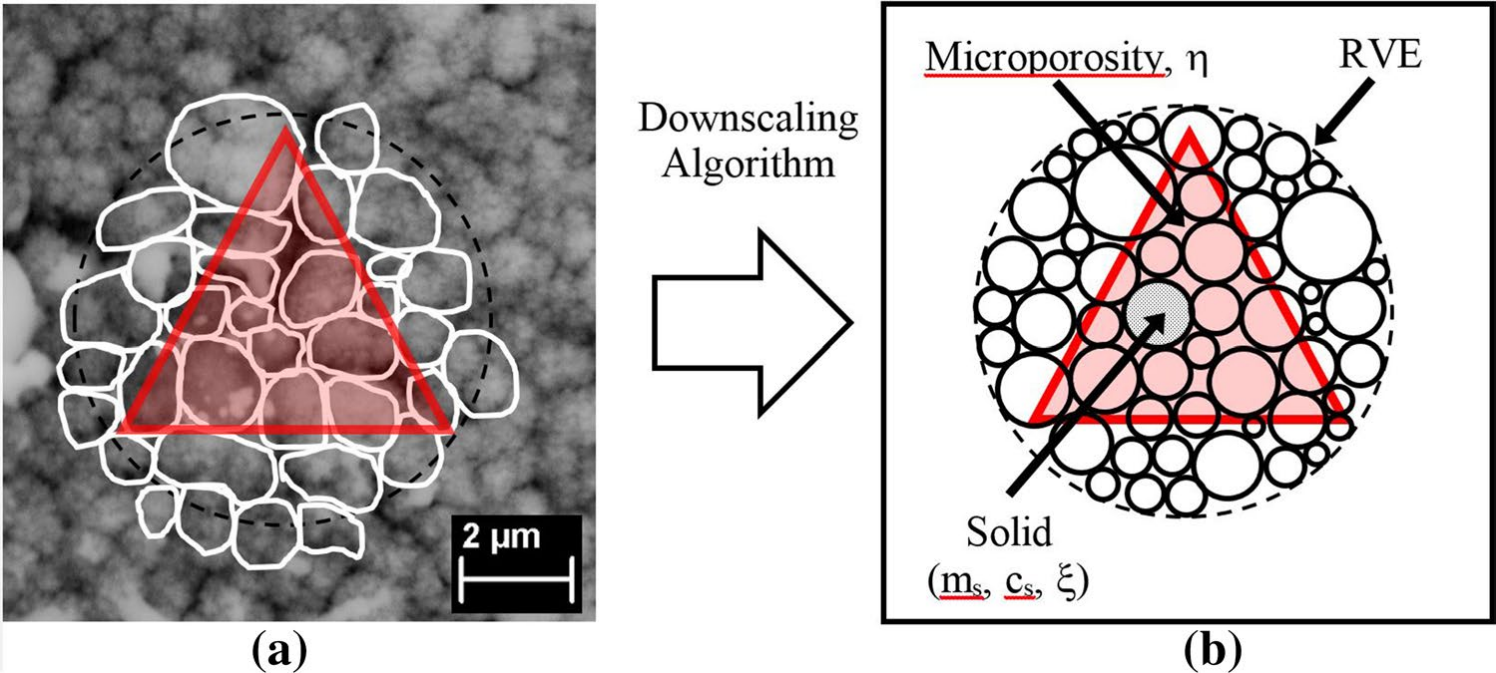
Indentation carried out on a solid-porous medium consists of solid particle and microporosity at microscale—Level (I). (**a**) Indentation surface (red) located within a RVE capturing response from both the solid particle and microporosity (**b**) Self-consistent, perfectly disordered, granular material model containing multiple phases solid particle with microporosity.

By choosing the right indentation depth, grid indentation technique^35^ has been shown to successfully allow access to the mechanical properties of individual phases within the heterogeneous multiphase material. By adopting the same approach, it is shown here that a single nanoindentation location carried out at the microscale (Level 1) on the thermal sprayed composite coating can access the properties of solid particles together with the microporosity. Figure 4 presents an illustration on how the microstructure of a thermal sprayed coating is simplified into a self-consistent and perfectly disordered solid-porous granular material model. The microporosity is represented by the packing density ($\eta$) which is the volumetric ratio of microporosity over the solid within a representative volumetric element (RVE). The solid particle can be represented by parameters including the elastic stiffness ($m_s$), cohesion ($c_s$) and friction coefficient ($\xi$). The micromechanics relationship between the microporosity and the solid particles can be described using the microporomechanics theory and methodology.

## Microporomechanics methodology

The solid-porosity model used in this work is built on the granular material, self-consistent, perfectly disordered micromechanical model that was incepted from the early by Jaeger and Sidney^36^. The corresponding anisotropic mechanical properties from the transversely isotropic model can be represented with a stress strain relationship in the form of elastic stiffness tensor (${\mathbb {C}}$)^37^ that is derived for a transversely isotropic model. Due to the symmetry of this model, the number of unknown in the stiffness tensor in matrix-vector form can be reduced to only five. These five unknown are outlined in Eq. (1)^32^ which can be used to describe the indentation modulus (*M*) for both the indentation directions.

1 $$\begin{aligned} \begin{aligned} M_3&=2\sqrt{\left( \frac{{\mathbb {C}}_{1111}C_{3333}-{\mathbb {C}}_{1133}^2}{{\mathbb {C}}_{1111}}\right) \left( \frac{1}{{\mathbb {C}}_{2323}}+ \frac{2}{\sqrt{{\mathbb {C}}_{1111}{\mathbb {C}}_{3333}}+ {\mathbb {C}}_{1133}}\right) ^{-1}} \\ M_1&=\sqrt{\sqrt{\frac{{\mathbb {C}}_{1111}}{{\mathbb {C}}_{3333}}} \left( \frac{{\mathbb {C}}_{1111}^2-\mathbb {{\mathbb {C}}}_{1122}^2}{{\mathbb {C}}_{1111}}\right) M_3} \end{aligned} \end{aligned}$$

where the ${\mathbb {C}}_{ijkl}$ is the stiffness tensor component in matrix-vector form. $M_1$ and $M_3$ are the indentation modulus in the longitudinal and transverse directions, which are corresponding to $x_1$/$x_2$ and $x_3$ axes in Fig. 1, respectively. Based on the microporomechanics analytical derivation^31,38,39^, the mechanical properties of solid particles have a distinct relationship with the change in microporosity, as shown in Eq. (2). The theoretical homogenised indentation modulus ($M_{hom}$) can be linked to the microporosity expressed in a scaling function and the solid particle’s elastic stiffness. Similarly, the theoretical homogenised hardness ($H_{hom}$) can be linked to the microporosity expressed in a scaling function and the plasticity behaviour of the solid particle, expressed in terms of cohesion ($c_s$) and friction coefficient ($\xi$).

2 $$\begin{aligned} \begin{aligned} {M_{hom}}&={m_s}\cdot \Pi _M\left( \frac{{\mathbb {C}}}{{\mathbb {C}}^s},\eta ,\eta _0=0.5\right) \\ {H_{hom}}&={c_s}\cdot \Pi _H(\xi ,\eta ,\eta _0=0.5) \end{aligned} \end{aligned}$$

where $\Pi _M$ and $\Pi _H$ are the dimensionless indentation modulus and hardness scaling function. The terms $m_s$ and $c_s$ are the solid particle’s elastic stiffness and cohesion. It is worth noted that the indentation modulus is directly related to the elasticity behaviour of the solid particle’s stiffness tensor (${\mathbb {C}}^s$) and the homogenised stiffness tensor (${\mathbb {C}}$) that represent the combined effect of solid particles with microporosity. It is when the packing density approaching unity, the homogenised stiffness tensor becomes the solid particle stiffness tensor, i.e. ${\mathbb {C}}^s=\lim \limits _{\eta \rightarrow 1} {\mathbb {C}}$. The cohesion ($c_s$) and friction coefficient ($\xi$) is adopted from conventional plasticity description as the yield criterion for a Drucker–Prager solid with pore spaces^39^. In this case, these two parameters are responsible for the determination of hardness which was previously recognised to have dominant plastic deformation for sharp indenter^40^. Both of these dimensionless scaling functions are bounded by the range of microporosity that is set from a percolation threshold ($\eta _0$) of 0.5–1 that is based on the nature of spheres packing study^41^. The indentation modulus and hardness scaling functions and their parameters have been investigated in previous literatures^42–44^ and adopted for this work, expressed as

3 $$\begin{aligned} \Pi _M\left( r_s,\eta ,\eta _0=0.5\right)= & {} \frac{\mathfrak {I}\left( 9\eta r_s+4\mathfrak {I}+3r_s\right) \left( 3r_s+4\right) }{4\left( 4\mathfrak {I}+3r_s\right) \left( 3r_s+1\right) } \end{aligned}$$

4 $$\begin{aligned} \Pi _H\left( \xi \right)= & {} \Pi _0\left[ 1+\left( 1-\eta \right) \xi - \left( d-e\eta \right) \xi ^2-\left( f-g\eta \right) \xi ^5\right] \end{aligned}$$

where $r_s$ is the ratio of solid particle’s bulk and shear modulus, $\mathfrak {I}$ is the composite shear modulus to solid particle’s shear modulus ratio, which is dependent on the $r_s$ and packing density, $\eta$. The above scaling functions are derived based on the assumption of isotropic condition. In order to include the effect of the transverse direction, this work adopts the Voigt-Reuss-Hill averaging technique^43^ to derive the ratio of solid bulk and shear modulus based on the indentation modulus in both directions (as presented in Eq. 1). More details of this approach are given in the authors’ previous work^20^.

At this point, it becomes evident that the theoretical homogenised indentation modulus and hardness cannot be solved deterministically because the local microporosity is unknown. In order to find out the local microporosity, a minimization process is required in order to match the theoretical microporosity onto each of the actual indentation experimental nanoindentation response. The minimization procedure is summarised in Eq. (5) which contains a two-steps minimisation approach with four sets of input data, i.e. longitudinal and transverse indentation modulus ($M_3$ and $M_1$) and hardness ($H_3$ and $H_1$), while *N* is the number of indentation locations. The first step in the minimisation process requires the minimisation of the elastic stiffness tensor so that solid particle’s stiffness can be determined. The second step of minimisation round includes the minimisation of the cohesion ($c_s$), friction coefficient ($\xi$) and the corresponding local packing density ($\eta$) that fulfil both the experimental indentation modulus and hardness in both directions.

5 $$\begin{aligned} \underset{c_s,\xi ,\eta }{min}\sum _{i=1}^{N}\underset{{\mathbb {C}}^s}{min} \sum _{i=1}^{N}\left[ \left( 1-\frac{M_{hom}^J}{M_i^J}\right) ^2+ \left( 1-\frac{H_{hom}^J}{H_i^J}\right) ^2\right] \end{aligned}$$

## Result and discussion

### Microstructure investigation

**Figure 5:**
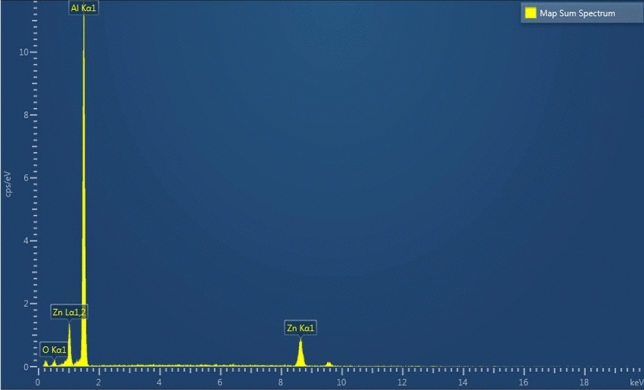
Aluminum–Zinc coating phase characterisation result by EDS.

**Figure 6:**
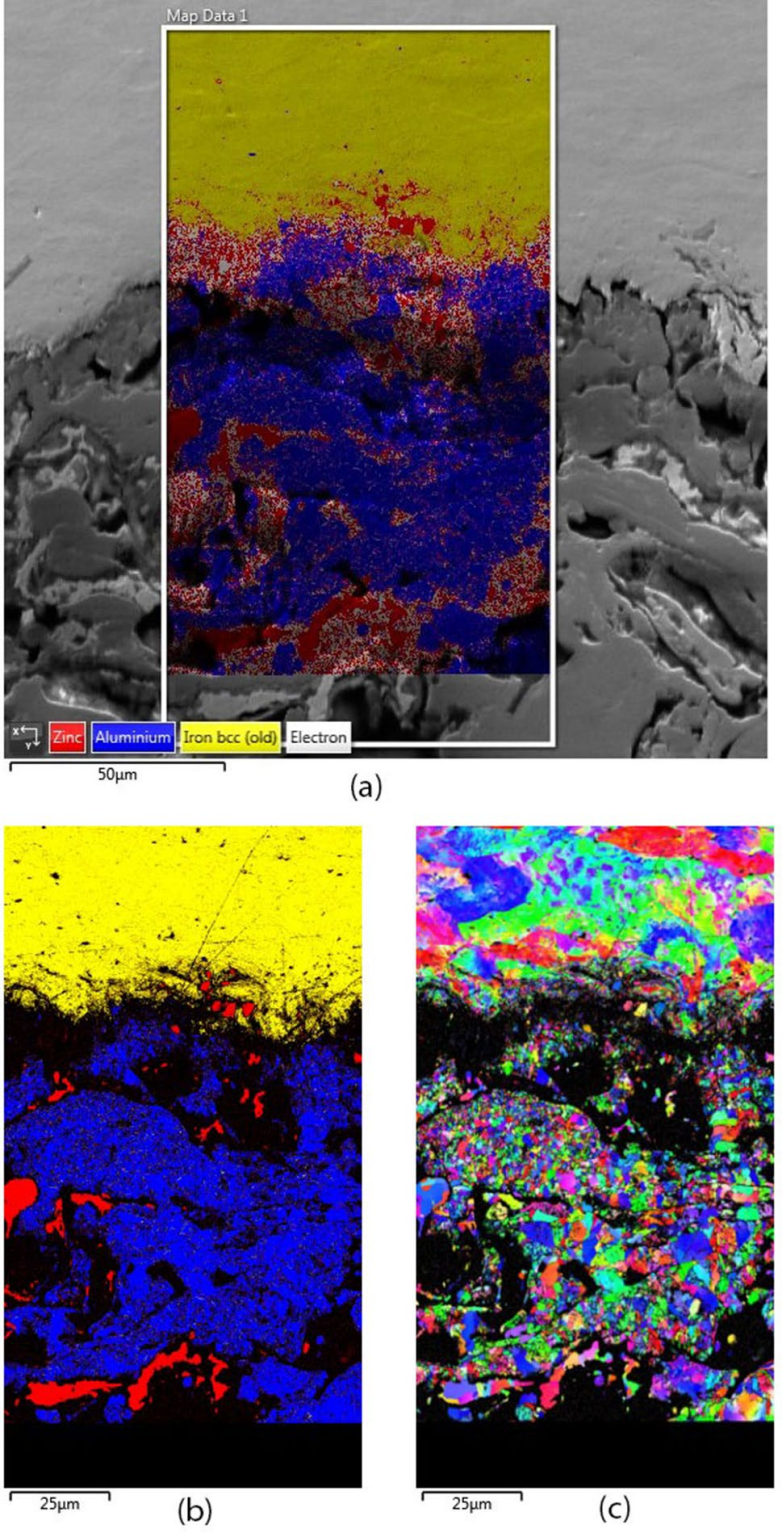
EBSD carried out on thermal sprayed Al–Zn coating cross section: (**a**) snapshot taken on coating with iron steel substrate (**b**) phase contrast showing aluminum (blue), zinc (red) and iron (yellow) (**c**) grain size and orientation.

The two-phase coating material is verified by scanning electron microscope (SEM) where the composition is shown in Fig. 5, which consists of aluminum and zinc. Furthermore, electron backscatter diffraction (EBSD) has been carried out on the coating cross-section to confirm the details of individual grains, as shown in Fig. 6. Figure 6a shows the snapshot taken at the composite aluminum and zinc coating interface with the iron steel substrate. Figure 6b shows that the contrast of the aluminum and zinc phases. The black areas in the image are the macroscale porosity represented by the voids and cracks occupied in between the deposited molten splats^1^. It could be observed that the aluminum and zinc formed both intra and inter composite microstructure^28^ that further added to the complexity of the nanoindentation results. The EBSD scan also reveals a relatively small grain size for the composite coating as a result of the vast extent of plastic deformations after exposure to high heat and kinetic pressure during the spraying process^29,30^. As a result, Fig. 6c shows the solid particle’s different grain orientation arrangement that is common to thermal sprayed coating and bulk metals and alloys^45,46^. It is worth noted that the effect of the grain orientation has not been taken into account in this study. Furthermore, the complexity in the microstructure shown in Fig. 6c has been simplified by adopting the self-consistent, perfectly disordered, granular material model mentioned in section 5. The mechanical properties are likely to vary in relative to one grain to another due to the different grain orientation. For this reason, the nanoindentation measurement is expected to obtain an overall response from indenting into multiple grains at a single location. Therefore, the mechanical properties determined from nanoindentation represent a statistical representation number, instead of a specific in-situ single grain’s property.

### Statistical deconvolution outcome

Using statistical deconvolution technique^5,31,42^, the indentation modulus, hardness and volume fraction of these phases for both directions are presented in Table 1. The results show that the indentation modulus and hardness are directional dependant. The range of anisotropic mechanical properties, which are determined based on finite element simulation and artificial neural network^20^, agrees well with the results reported in the literature^47,48^. The results also show anisotropic behaviour in both the indentation modulus and hardness. The observed anisotropic ratio for the indentation modulus obtained in this work is relatively small, which is about 20% for aluminum and 7% for zinc. This observation is in line with previous investigations^9,49^ on anisotropic behaviour in thin film and single crystal metal, which reported that the difference of the indentation modulus in the longitudinal and traverse directions obtained using nanoindentation is not apparent for a transversely isotropic material such as coating.

**Figure 7:**
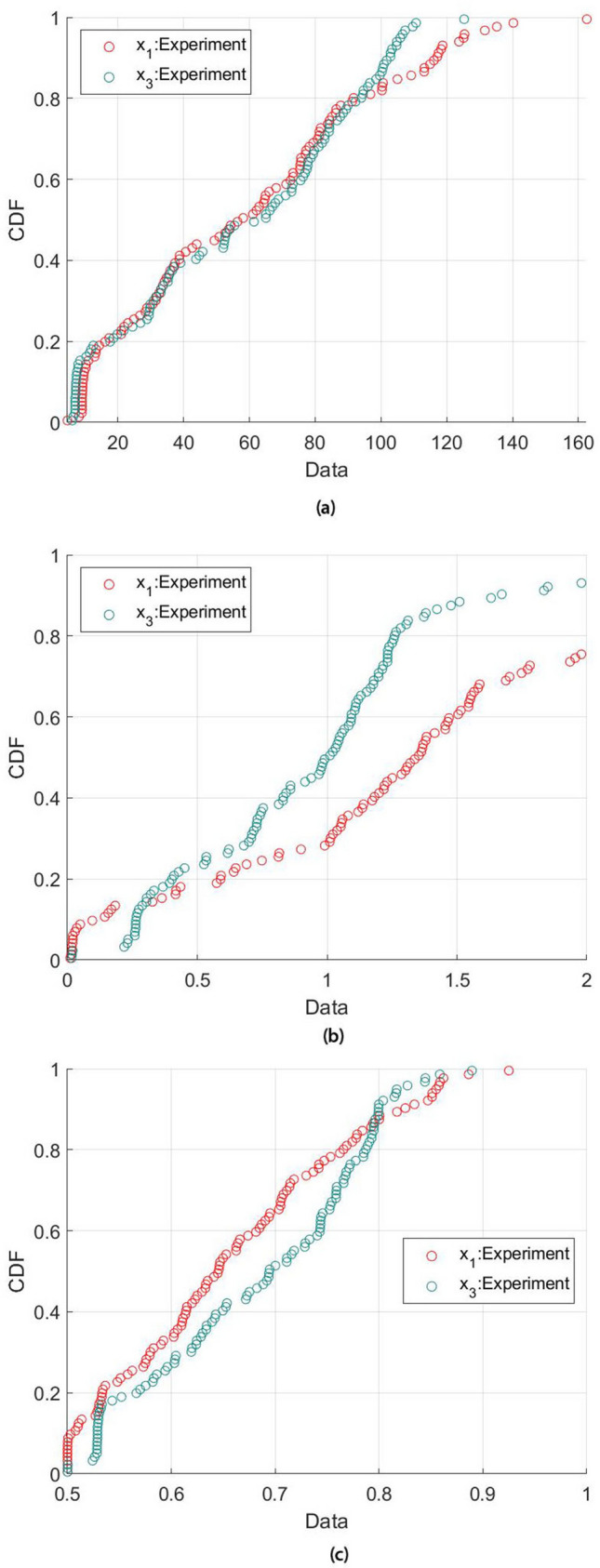
Cumulative distribution functions as a result from the deconvolution result (**a**) elastic modulus (**b**) hardness (**c**) packing density.

On the other hand, the observed anisotropic ratio for hardness is relatively larger in aluminum and zinc at 60% and 40% respectively. These observations are close to the ratio reported in existing literatures^49,50^. It is noted here that the indentation modulus and hardness of the thermal sprayed coating are relatively lower than the the values obtained from the bulk material^51^. The discrepancy is likely to be attributed by the presence of microporosity as theorised in the microporomechanics approach. The anisotropic behaviour can be clearly identified in the cumulative distribution functions, as shown in Fig. 7, where the indentation modulus, hardness, and packing density are literally different in both $x_1$ and $x_3$ directions.

**Table 1:** Deconvolution results of elastic modulus, hardness and packing density.

Direction	Phase	Elastic modulus (GPa)		Hardness (GPa)		Packing density (GPa)		Volume fraction %
		Mean	StD	Mean	StD	Mean	StD	
$x_1$	Aluminum	45.602	36.397	1.262	0.723	0.616	0.094	82
	Zinc	108.780	22.473	2.311	2.145	0.798	0.016	18
$x_3$	Aluminum	35.395	29.514	0.738	0.525	0.616	0.075	65
	Zinc	93.149	12.585	1.183	0.202	0.790	0.010	35

**Figure 8:**
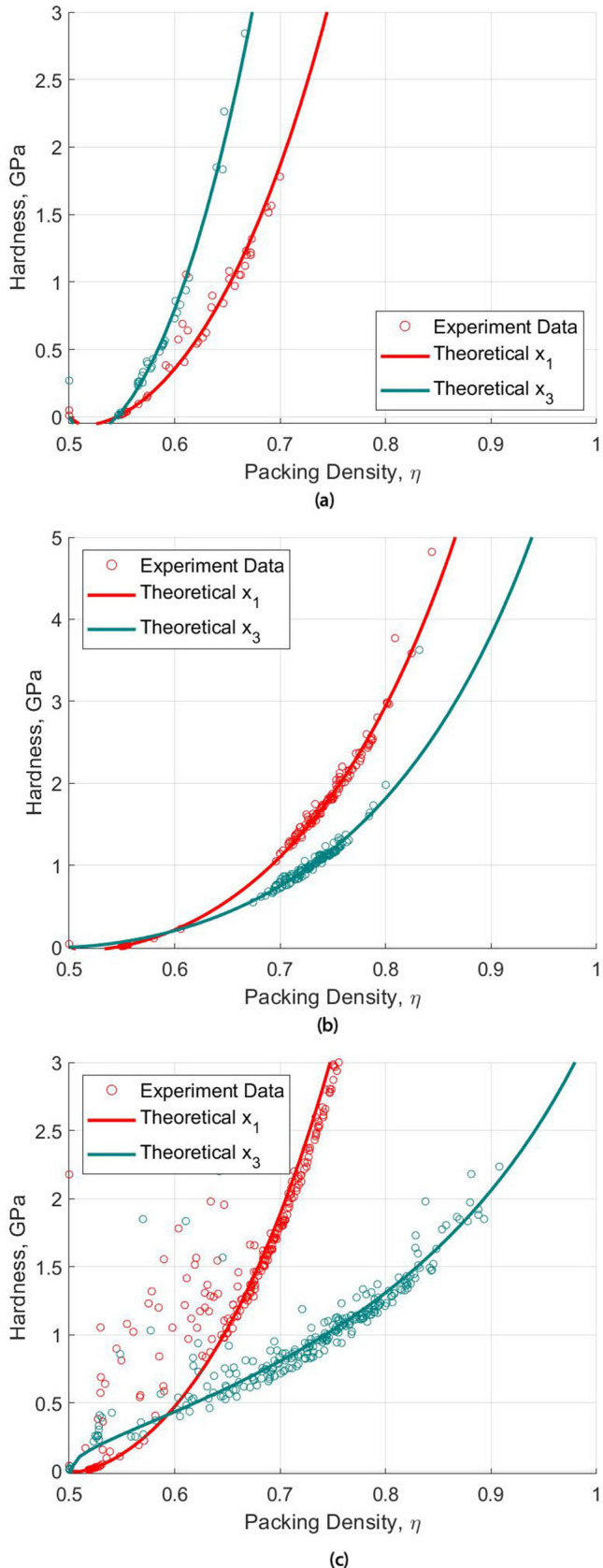
Scaling relationship of hardness with packing density (**a**) aluminum (**b**) zinc (**c**) Al–Zn composite.

The effect of microporosity on the hardness obtained by nanoindentation can be observed in Fig. 8. It is shown here that the hardness increases non-linearly with increasing packing density, i.e. reducing microporosity. It can be seen that the hardness is close to zero when the packing density is near and below the percolation threshold, $\eta \le 0.5$. This is because when the packing density is below this percolation threshold, the morphology structure is not stable and hence it is not possible to form a load path; thus, the hardness cannot be measured. Through the use of statistical deconvolution technique and based on the local packing density from the minimisation process, the characteristic mean packing density of the aluminum and zinc is 0.616 and 0.790, respectively, which is close to the highest spherical packing density of 0.74^36,52^ achievable for a face-centred cubic (FCC) and hexagonal close packed (HCP) system. The microporosity in terms of the packing density provides an alternative way to represent the imperfection in the packing of a grain structure, or more commonly known as the grain boundary defects, in the thermal sprayed coating.

Another key finding from the minimisation problem using the microporomechanics approach is the determination of the solid particle’s elastic stiffness tensor (${\mathbb {C}}$). Subsequently, the matrix-vector form of the elastic stiffness tensor can be used to determine the elastic modulus of the solid particle. The result is given in Tables 2 and 3 for the aluminum and zinc, respectively, in comparison with existing literature where elastic stiffness tensor is determined with alternative means including experimental, first principles and simulation using molecular dynamics^53–57^. The stiffness tensor defined using the combination of microporomechanics theory and experimental nanoindentation measurement is found to be within a reasonable range. It is observed here that the derived elastic modulus in the present work for both the aluminum and zinc indicates that the solid particle’s elasticity behaviour is close to isotropic given that the difference in both longitudinal and transverse direction is relatively small ($<\,4$%). Recalled from Eq. (2) that the indentation modulus is related to the solid particle’s stiffness and the microporosity. Given that the same microporosity has its effect on both indentation modulus and hardness, the observed isotropic elasticity behaviour in solid particle will infer that the anisotropic experimental measurement from nanoindentation is dominantly attributed from the solid particle’s plasticity parameters. In other words, the anisotropy in the thermal sprayed coating is found to be the result of the solid particle’s plasticity properties.

Table 4 outlines the properties of the solid particles as a result of the minimisation effort in matching the theoretical solid particle’s mechanical parameters with the experimental nanoindentation result. The difference in the solid particle’s stiffness in both directions are relatively small, which is 13% for aluminum and 6% for zinc. On the other hand, the plasticity properties, i.e. the cohesion ($c_s$) and friction coefficient ($\xi$), are shown to have a significant anisotropic result in both the longitudinal and transverse directions. Figure 8 also shows that the differences in the hardness for both directions are wider as the packing density increases. The difference in the cohesion ($c_s$) for aluminum is almost 5 folds in aluminum and 3.5 folds in zinc while the friction coefficient ($\xi$) differs in 30% and 70% in aluminum and zinc respectively. These observations reinforce the conclusion arrived earlier that the anisotropy in the coating is influenced dominantly by the material’s plastic yield criterion. In other words, the coating anisotropy is a result of the plastic yielding and post-yield hardening as the coating particles deposit and cool down following the spraying process. The results show that the dominant cohesion coincides with the spraying direction that can be explained by the kinetic pressure as a result of the splat deposition. This observation is in line with the literature ^48,58^ showing that the yield strength is dominant in the coating’s spraying direction.

**Table 2:** Elastic stiffness matrix and corresponding elastic modulus for aluminum.

Stiffness matrix component	Pascuet et al.^53^	Choudhary et. al.^54^	Present work
C1111	113.5	114.0	119.152
C1122	61.6	616.6	70.260
C1133	N.A.	N.A.	75.622
C2323	45.4	31.6	42.218
C3333	N.A.	N.A.	128.369
Elastic modulus, E1 (GPa)			65.742
Elastic modulus, E3 (GPa)			67.985

**Table 3:** Elastic stiffness matrix and corresponding elastic modulus for zinc.

Stiffness matrix component	Jang et. al.^55^	Dickel et. al.^56^	Romer et. al.^57^	Present work
C1111	133.43	177.0	179	170.336
C1122	47.02	34.8	38	53.305
C1133	41.51	52.8	55	51.075
C2323	34.14	45.9	46	104.476
C3333	122.42	68.5	69	162.417
Elastic modulus, E1 (GPa)	110.261	136.046	134.907	145.283
Elastic modulus, E3 (GPa)	103.322	42.175	41.120	139.089

**Table 4:** Solid state properties of aluminum and zinc.

Phase	Solid state properties	$x_1$	$x_3$
Aluminum	Stiffness, $m_s$ (GPa)	85.506	97.640
	Cohesion, $c_s$ (GPa)	0.182	0.929
	Friction coefficient	0.834	0.634
	Friction angle, $\xi$ (degree)	39.828	32.375
Zinc	Stiffness, $m_s$ (GPa)	166.913	177.05
	Cohesion, $c_s$ (GPa)	0.099	0.349
	Friction coefficient, $\xi$	0.834	0.481
	Friction angle (degree)	39.828	25.687

## Conclusion

This paper presents a methodology to investigate the anisotropic mechanical properties of the thermal arc sprayed composite (aluminum and zinc) coating using nanoindentation with the microporomechanics approach. This approach combines the microporomechanics theory with analytical tools, including statistical deconvolution technique, dimensional analysis, artificial neural network (ANN), and finite element simulation to determine the transversely anisotropic behaviour of the coating. Mass array experimental nanoindentation is carried out on the coating where the indentation modulus and hardness in the longitudinal direction (spraying direction) are obtained. The corresponding transverse nanoindentation responses are determined using ANN. Subsequently, a minimisation algorithm is used to determine the the solid particle’s mechanical properties, i.e. the stiffness, cohesion and friction coefficient, corresponding to the experimental nanoindentation response. The results show that the solid particle mechanical behaviour are related to the packing density, elasticity and plasticity properties The following conclusions are drawn based on the observation of the analysis outcomes:

- The statistical deconvolution technique can be used to identify the mechanical properties of individual coating’s phases and their packing density. Experimental nanoindentation shows that the coating exhibits relatively high anisotropic condition in the hardness compared to the indentation modulus.
- The packing density which represents the microporosity at microscale level (Level I) can be determined using the microporomechanics approach through a minimisation process. The hardness has a non-linear relationship with the packing density as predicted by the theoretical derivation of the dimensionless scaling function. The mean characteristic packing density is close to the theoretical highest spherical packing ratio for the face-centred cubic (FCC), and hexagonal close packed (HCP) system, i.e. aluminum and zinc respectively.
- The minimisation process between the experimental nanoindentation responses and the theoretical solid particle’s mechanical properties and local packing density results in the determination of the elastic stiffness tensor of the solid particle. The solid particle’s elastic modulus determined from the stiffness tensor is found to be close to isotropic, which suggests that the anisotropic condition in the coating is not related to the solid particle’s elasticity properties.
- The solid particle’s plasticity properties are found to have a significant anisotropic condition when compared to the elasticity properties, which are also corresponding to the state of packing density. It is observed that the coating’s anisotropic condition is dominantly affected by the cohesion and friction coefficient. These dominant plasticity properties correspond to the properties in the longitudinal (spraying) direction, which can be explained due to the relatively high kinetic pressure as a result of the deposition of splat in this direction.
